# Feature activation during word recognition: action, visual, and associative-semantic priming effects

**DOI:** 10.3389/fpsyg.2015.00659

**Published:** 2015-05-27

**Authors:** Kevin J. Y. Lam, Ton Dijkstra, Shirley-Ann Rueschemeyer

**Affiliations:** ^1^Donders Institute for Brain, Cognition and Behaviour, Radboud University NijmegenNijmegen, Netherlands; ^2^International Max Planck Research School for Language SciencesNijmegen, Netherlands; ^3^Department of Psychology, University of YorkYork, UK

**Keywords:** embodied language comprehension, feature activation, semantic priming, action priming, visual priming

## Abstract

Embodied theories of language postulate that language meaning is stored in modality-specific brain areas generally involved in perception and action in the real world. However, the temporal dynamics of the interaction between modality-specific information and lexical-semantic processing remain unclear. We investigated the relative timing at which two types of modality-specific information (action-based and visual-form information) contribute to lexical-semantic comprehension. To this end, we applied a behavioral priming paradigm in which prime and target words were related with respect to (1) action features, (2) visual features, or (3) semantically associative information. Using a Go/No-Go lexical decision task, priming effects were measured across four different inter-stimulus intervals (ISI = 100, 250, 400, and 1000 ms) to determine the relative time course of the different features. Notably, action priming effects were found in ISIs of 100, 250, and 1000 ms whereas a visual priming effect was seen only in the ISI of 1000 ms. Importantly, our data suggest that features follow different time courses of activation during word recognition. In this regard, feature activation is dynamic, measurable in specific time windows but not in others. Thus the current study (1) demonstrates how multiple ISIs can be used within an experiment to help chart the time course of feature activation and (2) provides new evidence for embodied theories of language.

## Introduction

One of the oldest issues in cognitive psychology concerns the mental representation of meaning. In the past decade, embodied theories of language, postulating that language meaning is stored in modality-specific brain areas, have gained in popularity and empirical support. For example, the meaning of the word “*grasp*” activates some of the neural areas involved in planning and performing everyday grasping actions (e.g., Hauk et al., 2004; Rueschemeyer et al., 2007), while comprehension of the word “*red*” entails activation of parts of the neural visual pathway (e.g., Simmons et al., 2007; van Dam et al., 2012). Nevertheless, despite much research important questions remain unanswered. One of these is when, and to what end, modality-specific information becomes activated during language comprehension.

In line with a general embodied framework, a number of behavioral studies have demonstrated that words with shared perceptual features prime each other. This indicates that the physical properties of an object in the real world influence how the word denoting the object is processed. For example, words referring to objects with similar shapes, such as *pizza* and *coin*, prime each other (Schreuder et al., 1984; Pecher et al., 1998), as do words referring to objects with shared manipulation features such as *typewriter* and *piano* (Myung et al., 2006). Note that in both of these examples, participants showed priming (which is interpreted as facilitation of processing) for words with shared perceptual or action features, even in the absence of any obvious conceptual or semantic relationship (see McNamara, 2005 for an in-depth treatment).

Neuroimaging evidence such as those reported by Kiefer et al. (2011) using EEG and fMRI further substantiate the results above. Participants saw a prime presented either as a word or as a picture followed by a target picture and were asked to name both stimuli when cued. Pairs were either congruent (*pliers*–*nutcracker*) or incongruent (*pliers*–*horseshoe*) with regard to the implied action. Most notably, pictures primes elicited early (N1) and late (N400) priming effects; word primes, by contrast, showed effects only later in the N400 component. The authors interpreted the finding as evidence of two stages of priming effects: fast and slow activation of action features with pictures, but slow activation with words. Specifically, the authors argued that pictures make certain features more salient, therefore activating more detailed representations which may also lead to earlier activation. Word stimuli appear less suitable to generate early action priming effects, at least when manipulations of congruency are employed to induce priming effects.

Other experimental methods have also been used to test for the activation of visual information (i.e., information about the visual form of an object in the real world) in conjunction with word processing. In an eye-tracking study (Yee et al., 2011), participants heard a spoken word and saw pictures of four objects on a screen in a given trial. In this visual world paradigm, participants identified the picture that best matched the spoken word. Notably, participants spent significantly longer looking at distractor items with a visual form matching that of the object denoted by the spoken word. For example, when participants heard “*frisbee*”, they looked significantly longer at a picture of a pizza (both objects are round) than to a linguistically matched control with no shared perceptual features (e.g., a thimble). Interestingly, this effect appeared only if participants had a relatively short amount of time to explore the visual scene (1000 ms); the effect was not present when the visual scene was presented for a longer time period (2000 ms). The authors argue that effects of visual form are seen early in word and object identification but decay over time.

Altogether, the reviewed studies suggest that different aspects of a word's referent (i.e., the object's features) can be independently activated, as evidenced by action and visual priming effects. Nonetheless, studies make the implicit assumption that feature activation is constant and stable over time, as is evident from their use of a single inter-stimulus interval (ISI) or stimulus-onset asynchrony (SOA) value in most (priming) experiments. Interestingly, in Yee et al. (2011), the authors reported a possible decay of visual features over time which was determined by comparing two ISIs. In Kiefer et al. (2011), the authors proposed fast and slow feature activation as a function of stimuli type, but it is unclear if the same conclusion will hold if at least another ISI was tested for a comparison. Still, unlike the commonly held assumption, the authors of both studies assume that the time course of feature activation is dynamic.

This focus on the timing aspect is non-trivial, especially when considered alongside the discussion of embodied theories of language. Some authors have demonstrated very fast (160–250 ms after word onset) and automatic (activation even when attention is diverted away) activation of sensorimotor information, taking this as evidence for the integral role of such information in the word's representation (Pulvermüller et al., 2001; Hauk and Pulvermüller, 2004; for a review, see Pulvermüller, 2005). However, other researchers have come to slightly different positions with respect to timing. In the Language as Situated Simulation (LASS) model, Barsalou and colleagues have claimed that perceptual and action information, while being an integral part of conceptual knowledge, are activated relatively late during language comprehension (Solomon and Barsalou, 2004; Barsalou, 2008; Barsalou et al., 2008). The slower reaction times for property verification judgements reflect the need for participants to activate deep, perceptually-based conceptual knowledge, because quickly accessed language-based relationships will not suffice to perform the task.

The Symbol Interdependency Hypothesis (SIH; Louwerse, 2011), too, claims that perceptual simulations play a greater role later on and reflect more detailed representations but, unlike the LASS model (Barsalou, 2008), it emphasizes the symbolic (linguistic) rather than the embodied (modality-specific) aspect of linguistic processing. For example, Louwerse showed that the results of a previous iconicity study (Zwaan and Yaxley, 2003) which was interpreted as support for embodied representations, could actually be accounted for predominantly by linguistic frequency. In other words, SIH argues that perceptual simulations can be traced back to language itself. The symbolic aspect serves to create underspecified representations quickly for good-enough comprehension whereas the embodied aspect goes further when full and deep comprehension is needed by relying on embodied relations already encoded into language. In sum, LASS and SIH make similar proposals; only the relative importance of each component differs whereby the task dictates which component is more or less relevant. Both theories claim that perceptual features are time-consuming and resource-hungry.

In the current behavioral priming experiment, we aim to disentangle some of the issues surrounding the time course along which different types of perceptual and motor information are activated during word comprehension. Firstly, we focus on action and visual features because previous research has shown the two features to be highly relevant in word processing. More importantly, action and visual features have not been directly compared within one study to determine whether they each have unique time courses. Furthermore, other studies (e.g., Wheatley et al., 2005) that suggest relative importance of different features for the conceptual representations of objects are another source for our hypothesis. Based on previous literature (e.g., Schreuder et al., 1984; Pecher et al., 1998; Myung et al., 2006), we hypothesize that both feature-based action and visual priming should show effects similar in direction to those seen for associative semantic priming. That is, word pairs related along action and visual features should show facilitation of reaction times.

Secondly, we use the Go/No-Go task in combination with lexical decision. In the current experiment, participants are instructed to respond with a button press when a stimulus pair consists of words (“Go”); otherwise, they did not need to respond (“No-Go”). Notably, some authors have used the task to examine time-course related issues in language processing (e.g., van Turennout et al., 1997, 1998, 1999). Nevertheless, we included associative-semantically related stimuli to elicit standard semantic priming effects as a verification measure (see Gomez et al., 2007 for a comparison of the Go/No-Go and standard two-choice tasks).

Thirdly, we systematically vary the time between presentation of the prime word and the target word; that is, the ISI, which is the interval between the offset of the prime and the onset of the target. Participants in the pilot phase reported that they could not always identify the prime and target stimuli if a presentation duration of less than 400 ms was used (mean word length no less than 9.5 letters; see Supplementary Material for complete listing). Consequently, we used a fixed prime and target duration of 400 ms and varied the ISIs accordingly. The ISI factor is thus a manipulation of preview time between prime and target word presentation to determine the relative timing of and processing differences between different features. We assume that activation is a dynamic process, thus there is likely no single ISI value that can capture all features; the use of multiple ISI values therefore is intended to sample feature activation over time (see Moss et al., 1995; Hauk et al., 2012 for similar arguments). Previous relevant priming studies (Myung et al., 2006; Kiefer et al., 2011) have used ISIs of 50 and 70 ms, with SOAs ranging between 370 and 1250 ms. In line with those earlier studies, we employed three ISIs in 150 ms-increments: 100, 250, and 400 ms (corresponding to SOAs of 500, 650, and 800 ms, respectively). The fourth ISI of 1000 ms (equal to an SOA of 1400 ms) serves as a long interval in which we expect the greatest modulation of effects to occur.

In summary, we investigate priming in three distinct conditions: (1) associative semantic priming (e.g., *bolt–screwdriver*), (2) feature-based action priming (e.g., *housekey–screwdriver)*, and (3) feature-based visual form priming (e.g., *soldering iron–screwdriver*). By including three priming conditions within one experimental design, we investigate whether feature-based action and visual priming produce effects directly comparable with associative semantic priming. More importantly, by looking at priming at four ISIs we assess how long after presentation of a prime word, specific types of information become available in order to affect comprehension of the target word. In this manner, we can draw conclusions about the relative timing of different types of feature-based semantic knowledge. Different embodied theories of language predict such effects at different time intervals: strong embodied theories (e.g., Pulvermüller, 2005; Pulvermüller and Fadiga, 2010) predict effects in the early phase, but moderate and disembodied theories (e.g., Mahon and Caramazza, 2008) in the late phase. Hybrid theories such as LASS (e.g., Barsalou, 2008) and SIH (Louwerse, 2011) allow the involvement of both language-based and perceptual-based information, with more or less emphasis on either depending on the task. The current study will provide detailed timing information to help adjudicate between the competing theories.

## Materials and methods

### Participants

One hundred and seventy-six right-handed native German speakers aged 18–25 years (136 females; mean age = 21 years) with normal or corrected-to-normal vision were recruited within the Radboud University Nijmegen. Participants were assigned to one of the four inter-stimulus interval (ISI) groups, consisting of 44 participants each. Participants gave informed consent and were offered course credit or monetary compensation. This study was approved by the local Nijmegen Ethical Committee of the Faculty of Social Sciences (ECG2012-2711-05).

### Stimulus materials

German words denoting familiar tools or manipulable objects were used either as prime or target words. Each of the 24 target words was paired with four prime words corresponding to the four prime conditions (see sample stimuli in Table 1; full stimulus materials in Supplementary Material): (1) semantically related, (2) action-related, (3) visual-related, and (4) unrelated. In the semantically related condition, the prime and target pair denoted related objects by association (e.g., *bolt–screwdriver*) and had no action and visual relatedness. In the action-related condition, the prime and target pair denoted objects that are used in a similar manner but do not have any semantic or visual relatedness (e.g., *housekey–screwdriver*). Also, all actions implied by these objects are restricted to the hands or arms. In the visual-related condition, the prime and target pair denoted objects similar in form or appearance but did not share any semantic or action relatedness (e.g., *soldering iron–screwdriver*). Finally, the prime and target pair in the unrelated condition denoted objects that shared none of the above relationships (e.g., *charger–screwdriver*).

**Table 1:** **Sample primes from the four conditions paired with the same target in German with their corresponding English translations**.

	**Prime word**	**Target word**
Unrelated	Ladegerät *“charger”*	Schraubenzieher *“screwdriver”*
Semantic	Dübel *“bolt”*	
Action	Haustürschlüssel *“housekey”*	
Visual	Lötkolben *“soldering iron”*	

*See supplementary materials for full set*.

A norming study using a new selection of participants (*n* = 10) confirmed our manipulations (see Supplementary Material). For all comparisons of interest, words were matched for length and frequency (see Supplementary Material) using the SUBTLEX-DE database (Brysbaert et al., 2011). Also, 24 pseudowords were added from a pseudoword generator (Keuleers and Brysbaert, 2010) to serve as catch trials in the Go/No-Go lexical decision task, described below.

### Design

Participants were presented with a total of 140 trials: 96 critical trials containing 24 target words paired with four different prime words, 24 catch trials containing one or two pseudowords, and another 20 filler trials similar to critical and catch trials. The trials were divided into four blocks of 35 trials each, with five dummy trials at the beginning of each block. Crucially, target words appeared only once per block and lists were pseudo-randomized to ensure that no more than three consecutive trials were from the same condition. In result, four lists were generated and one version was randomly assigned to each participant.

### Procedure

Participants sat approximately 80 cm in front of the computer screen. Button presses were recorded from a response box. The start of a trial was indicated by an asterisk positioned at the center for 2000 ms. Next, prime and target words were each presented for 400 ms; the interval of the intervening blank screen—the inter-stimulus interval (ISI)—was 100, 250, 400, or 1000 ms. A black blank screen was presented for an inter-trial interval of 2000 ms.

Participants were instructed to press the response button with their right index finger whenever a trial consisted only of German words (i.e., both prime and target words). Otherwise, they were instructed to withhold their response—thus, catch trials (containing pseudowords) did not require a button press. A short break was given between blocks of trials. Participants were first presented with a practice block of 12 trials that did not contain any critical stimuli but reflected the experimental conditions. In total, each version of the experiment lasted about 20 min.

## Results

Participants were excluded if (1) their overall mean reaction times (RTs) exceeded 800 ms, and if (2) the d-prime scores of at least three conditions were less than 2.9 out of a maximal possible score of 4.7. Of the remaining data, we excluded incorrect trials and trials containing RTs faster than 250 ms and slower than 1800 ms, as well as those slower than 2.5 standard deviations of a participant's mean. This resulted in the removal of 3% trials. Priming scores were calculated by subtracting each of the three conditions (Semantic, Action, Visual) from the Unrelated condition.

For the F_1_ analyses, subject-based means were then submitted to a Two-Way Condition (Semantic, Action, Visual) × ISI (100, 250, 400, 1000-ms) ANOVA with Condition as a within-subject variable and ISI as a between-subject variable. For the F_2_ analyses, item-based means were submitted to a Two-Way Condition × ISI ANOVA with Condition and ISI both as within-subject variables. We also report complementary F_1_ and F_2_ analyses using only Action and Visual for the Condition factor to verify that the two main effects of interest indeed differ in time course. We report Greenhouse-Geisser corrected *p*-values whenever the sphericity assumption is violated.

Within each ISI group, paired samples *t*-tests were conducted for the three critical pairwise comparisons. All *p*-values resulting from the *t*-tests have been controlled for multiple comparisons using the Benjamini and Hochberg False Discovery Rate (FDR) procedure (Benjamini and Hochberg, 1995). Effect sizes reported reflect Cohen's *d* using pooled variance. See Table 2 for an overview of mean RTs.

**Table 2:** **The sample size, mean reaction times, and standard deviation values (within parentheses) of the four conditions across the four inter-stimulus interval manipulations**.

	**100 ms**	**250 ms**	**400 ms**	**1000 ms**
	***n* = 34**	***n* = 35**	***n* = 41**	***n* = 37**
Unrelated	547 (87)	560 (71)	558 (87)	553 (67)
Semantic	525 (84)	543 (68)	542 (87)	534 (67)
Action	536 (69)	550 (70)	554 (84)	542 (70)
Visual	552 (95)	554 (72)	553 (83)	543 (70)

### Interactions and main effects

A summary of the ANOVA analyses is shown in Table 3. Table 4 lists the priming scores of the three conditions; asterisks denote significant effects at FDR-corrected *p*-values < 0.05. The presence of a (nearly) significant interaction between Condition and ISI allowed us to consider the effects in each of the ISIs separately.

**Table 3:** **The ANOVA summary of F_1_ and F_2_ results using all three priming conditions and only the two main conditions of interest**.

	**Condition (semantic, action, visual)**	**Condition (action, visual)**
Condition x ISI	*F*_1(5.691, 271.255)_ = 2.079; *p* =0.059 *F*_2(6, 138)_ = 2.667; *p* = 0.018	*F*_1(3, 143)_ = 2.309; *p* = 0.079 *F*_2(3, 69)_ = 3.321; *p* = 0.025
Condition	*F*_1(2, 286)_ = 19.261; *p* = 0.000 *F*_2(2, 46)_ = 5.927; *p* = 0.005	*F*_1(1, 143)_ = 3.794; *p* = 0.053 *F*_2(1, 23)_ = 0.776; *p* = 0.388
ISI	*F*_1(3, 143)_ = 0.366; *p* = 0.778 *F*_2(3, 69)_ = 0.187; *p* = 0.905	*F*_1(3, 143)_ = 0.684; *p* = 0.563 *F*_2(3, 69)_ = 0.515; *p* = 0.673

**Table 4:** **Priming scores (in ms) of the three conditions and standard error of differences values (within parentheses) across the four inter-stimulus interval manipulations**.

	**Semantic**	**Action**	**Visual**
100 ms	22 (4.1)^*^	11 (5.6)^*^	−5 (5.9)
250 ms	17 (4.5)^*^	9 (4.4)^*^	6 (4.8)
400 ms	16 (4.4)^*^	4 (4.7)	5 (4.2)
1000 ms	19 (4.2)^*^	12 (4.8)^*^	10 (4.7)^*^

*Asterisks denote significant effects at FDR-corrected p-values < 0.05*.

### ISI = 100 ms

A statistically reliable semantic priming effect was present at this ISI: Mean RTs were faster to semantically related target words (525 ms) than to unrelated target words (547 ms), *t*_(33)_ = 5.33, *p* < 0.01, *d* = 0.253. An action priming effect was also statistically reliable: Mean RTs were faster to action-related target words (536 ms) relative to unrelated target words, *t*_(33)_ = 2.00, *p* < 0.05, *d* = 0.143. There was no statistically reliable visual priming effect, however. Mean RTs to visual-related target words (552 ms) were not distinguishable from those to unrelated target words, *t*_(33)_ = −0.91, *p* = 0.19, *d* = 0.060.

### ISI = 250 ms

A similar pattern of results as for ISI = 100 ms was found. A statistically reliable semantic priming effect was present: Mean RTs were significantly faster to semantically related target words (543 ms) than to unrelated target words (560 ms), *t*_(34)_ = 3.75, *p* < 0.01, *d* = 0.242. A statistically significant action priming effect indicated that mean RTs were significantly faster to action-related target words (550 ms) than to unrelated target words, *t*_(34)_ = 2.09, *p* < 0.05, *d* = 0.132. However, the visual priming effect was not statistically reliable: Mean RTs were not significantly faster to visual-related target words (554 ms) than to unrelated target words, *t*_(34)_ = 1.25, *p* = 0.12, *d* = 0.086.

### ISI = 400 ms

Only a semantic priming effect was obtained: Mean RTs were significantly faster to semantically related target words (542 ms) than to unrelated target words (558 ms), *t*_(40)_ = 3.51, *p* < 0.05, *d* = 0.178. Action and visual priming effects, however, were not statistically reliable. Mean RTs were not significantly faster to action-related target words (554 ms) than to unrelated target words, *t*_(40)_ = 0.81, *p* = 0.22, *d* = 0.045. Similarly, mean RTs were not significantly faster to visual-related target words (553 ms) than to unrelated target words, *t*_(40)_ = 1.23, *p* = 0.17, *d* = 0.061.

### ISI = 1000 ms

All three priming effects were statistically significant. Mean RTs were significantly faster to semantically related target words (534 ms) than to unrelated target words (553 ms), *t*_(36)_ = 4.55, *p* < 0.01, *d* = 0.283. Mean RTs were faster to action-related target words (542 ms) than to unrelated target words, *t*_(36)_ = 2.39, *p* < 0.05, *d* = 0.169. Finally, mean RTs were faster to visual-related target words (543 ms) than to unrelated target words, *t*_(36)_ = 2.14, *p* < 0.05, *d* = 0.146.

## Discussion

In this study, we investigated the time course of activation for different modality-specific features using a Go/No-Go priming paradigm with varying inter-stimulus intervals (ISIs). Four groups of participants performed lexical decisions to word pairs from three priming conditions: (1) associative semantic priming (e.g., *bolt–screwdriver*), (2) feature-based action priming (e.g., *housekey–screwdriver*), (3) feature-based visual priming (e.g., *soldering iron–screwdriver*), and we compared these to a fourth unrelated condition (e.g., *charger–screwdriver*). By varying the amount of time between presentation of the prime word and of the target word (i.e., ISI), we assessed how soon the activation of semantically relevant (i.e., feature-based) information became effectively available after prime word presentation.

Our results show that feature-based information present in the prime word facilitates recognition of subsequent target words (i.e., priming takes place). Importantly, the relative timing at which feature-based information becomes activated varies between modalities. Feature-based action relationships elicited priming effects at ISIs of 100, 250, and 1000 ms. Feature-based visual relationships, by contrast, elicited priming effects only at ISI of 1000 ms. Unlike both feature-based relationships, associative semantic relationships elicited consistent priming effects across all four ISIs.

In the following, we will first discuss the time course of activation of semantic, action, and visual features individually. As noted in the introduction, by varying the ISI (preview time), we can determine the relative timing of and processing differences between different features. We will argue that the finding of different time course of activation for different modality-specific features requires a reassessment of current opposing views on embodied representations, moving to views that highlight the flexible recruitment of feature activations (e.g., Hoenig et al., 2008; Kiefer and Pulvermüller, 2012) and a combination of amodal and embodied representations (e.g., Barsalou, 2008; Louwerse, 2011).

### Associative semantic priming effects are activated at all ISIs

We observed associative semantic priming effects at all four ISIs. These effects show that the experiment is sensitive to our manipulations and able to elicit priming effects at all four intervals tested. The findings agree with the literature on semantic priming wherein reports of semantic priming effects have been shown using very short and very long ISIs (e.g., Perea and Gotor, 1997; Hutchison et al., 2001; Perea and Rosa, 2002; Chiarello et al., 2003; see Hutchison, 2003 for a review).

### Different time courses of activation: action precedes visual feature activation

The results show that words referring to manipulable objects can indeed elicit action priming effects, as reported in the object representation literature (e.g., Ellis and Tucker, 2000; for a review, see Martin, 2007). In a similar action priming study (Myung et al., 2006 Experiment 1), participant made lexical decisions to primes and targets (e.g., piano–typewriter) presented over headphones. Another study (Kiefer et al., 2011) showed that picture targets preceded by word primes elicited effects relatively late in processing, namely in the N400 time window. By contrast, picture targets preceded by picture primes showed effects sooner in the N1 time window. Kiefer and colleagues argue that pictorial stimuli make certain features more salient, thus generating more detailed representations. However, retrieving more detailed representations does not necessarily lead to activation of a feature earlier in time, because such retrieval may be more time-consuming and effortful. Regardless, our results demonstrate that visually presented word pairs can elicit action priming effects in time windows subsequent to the N400.

We also observed priming effects of visually related word pairs in the longest ISI of 1000 ms. Unlike action features, visual features do not appear to be activated as quickly as action features. Seen alongside the semantic and action priming results, this suggests that different features may have different activation profiles.

Certain visual features may be particularly difficult to elicit using word stimuli. Using pairs of perceptually related stimuli which shared shape or color features, Schreuder and colleagues (Schreuder et al., 1984; Flores d'Arcais et al., 1985) reported priming effects using the lexical decision task. Subsequent studies, however, failed to replicate these effects unless these features were made explicit for the task, such as the use of a preceding activation task (Pecher et al., 1998; stimulus-onset asynchrony, SOA = 350 ms, ISI = 50 ms).

A possible clarifying factor is that the perceptual priming effect in Schreuder et al. (1984; also see Flores d'Arcais et al., 1985) is not strictly visual priming in the sense used here and elsewhere (e.g., Kellenbach et al., 2000). Their perceptual condition was composed of visually–(primarily) and color-related stimuli. Though color-related items made up a small part of the stimuli, the effects may have largely originated from these items. Color has been shown to be a prominent component of an object's representation, more so than action features for certain classes of object nouns (e.g., van Dam et al., 2012). Similarly, the perceptual stimuli used in Pecher et al. (1998) differ from our stimuli in that they consisted of nouns referring to a range of categories like food, body part, animals, etc., and could thus have confounded the results.

Using pictorial stimuli as targets, a recent study has indeed reported early visual effects (Yee et al., 2011) but, as is the case in the Kiefer et al. (2011) study with action features and pictorial targets, these early effects may appear sooner when pictorial stimuli are used. There is suggestive evidence that pictures are processed faster and yield larger effects than words across a range of tasks (e.g., Glaser, 1992). Future studies are needed to explicitly test different stimulus types using multiple ISIs, or even a combination of different experimental methods (e.g., RT and EEG as in Kellenbach et al., 2000; ISI = 150 ms).

### Implications for embodied theories of language

In the Language and Situated Simulation (LASS) theory, Barsalou et al. (2008) proposed that linguistic and situated simulation systems interact continuously. The fast linguistic system processes linguistic forms, not meaning, and thus allows for quick and effective performance in many cases. Meaning is derived by the slower and more central simulation system when the task at hand requires the retrieval of detailed representations. Similarly, the Symbol Interdependency Hypothesis (SIH; Louwerse, 2011) makes explicit predictions in terms of early and late contributions of symbolic (linguistic) and embodied (simulation) representations. Unlike LASS, SIH placess greater emphasis on linguistic representations because “language encodes perceptual information” (Louwerse, 2011, p. 279); thus meaning can be derived already from linguistic representations.

The current findings very broadly support the distinction between early and late stages of feature activation described by both LASS and SIH. Although both theories attribute early and late effects to different systems (linguistic and simulation, respectively), our results suggest that both systems are in play already at an ISI of 100 ms (equal to an SOA of 500 ms). Associative semantic priming effects across all ISIs show that the linguistic system is continuously activated, whereas action and visual priming effects at different ISIs show differential involvement of the simulation system. We attribute action and visual priming effects to the simulation system because it is unclear how statistical interdependencies which drive the linguistic system can pick up, for example, shared manipulation features between “*housekey”* and “*screwdriver”* that do not co-occur to any regularity. In our view, both associative semantic and action priming effects demonstrate the parallel activation of the linguistic and simulation systems (but see Louwerse and Hutchinson, 2012; Hutchinson et al., 2014), thus demonstrating the fast and dynamic nature of the overall conceptual system.

From a theoretical standpoint, the current findings can be interpreted as support for both LASS and SIH. Whether meaning is derived from (or, “resides” in) either the linguistic or simulation system requires further experimentation, but we suspect that both systems are involved and interdependent through flexible recruitment of feature activations (e.g., Hoenig et al., 2008; Kiefer and Pulvermüller, 2012) and a combination of amodal and embodied representations (e.g., Barsalou, 2008; Louwerse, 2011). Indeed, we argue that a more beneficial pursuit for embodied theories of language is to describe how the time course of feature activation relates to the way knowledge is acquired, represented, and retrieved given that these theories emphasize how conceptual representations are deeply rooted in interactions of the body and the world. Furthermore, future studies should chart changes in time courses as a function of task and context to clarify how the brain makes available different kinds of information according to present needs (e.g., Hoenig et al., 2008).

## Conclusions

Our results support the following account of the time course of visual word recognition. Feature activation is both fast and slow (e.g., Zwaan, 2003; Pulvermüller et al., 2005; Barsalou et al., 2008; Louwerse, 2011), and once a feature is activated, it can affect relatively early aspects of target word recognition (i.e., priming effects *do* occur). Different features have different time courses, and the relative timing of each feature is informative about the role the feature plays in the word representation of the object. Much empirical support has been offered in support of either the early or late activation of embodied representations (e.g., Glenberg and Kaschak, 2002; Louwerse and Jeuniaux, 2010; for a review, see Meteyard et al., 2010), but by comparing different ISIs within one study, we were able to determine that different modality-specific information is activated at different time points during visual word recognition.

### Conflict of interest statement

The authors declare that the research was conducted in the absence of any commercial or financial relationships that could be construed as a potential conflict of interest.
