# Supplementary material for: Feature activation during word recognition: action, visual, and associative-semantic priming effects
Source: Front Psychol. 2015 May 27;6:659. doi: 10.3389/fpsyg.2015.00659 (PMC4444743; doi:10.3389/fpsyg.2015.00659)
Supplement: Supplementary file 1 [file DataSheet1.DOCX]

**Appendix 1**. Stimuli for the four critical prime conditions and the target words.

| **prime** | | | | **target** | |
| --- | --- | --- | --- | --- | --- |
| **unrelated** | **semantic** | **action** | **visual** | |  |
| Salatschüssel  *salad bowl* | Fischnetz  *fishing net* | Peitsche  *whip* | Autoantenne  *radio antenna* | | Angelrute  *fishing rod* |
| Gürtelschnalle  *belt buckle* | Gummistiefel  *rainboots* | Fahrradluftpumpe  *bike pump* | Heizpilz  *patio heater* | | Regenschirm  *umbrella* |
| Klappstuhl  *folding chair* | Benzinkanister  *gas tank* | Nussknacker  *nutcracker* | Spielautomat  *slot machine* | | Zapfsäule  *gas pump* |
| Alufolie  *aluminum foil* | Dieselmotor  *diesel engine* | Tresortür  *vault door* | Autoreifen  *car tires* | | Steuerrad  *steering wheel* |
| Mülltüte  *trashbag* | Notizbuch  *notebook* | Essstäbchen  *chopsticks* | Laserpointer  *laser pointer* | | Kugelschreiber  *ballpoint pen* |
| Brechstange  *crowbar* | Reflektor  *reflector* | Fernbedienung  *remote control* | Mikrophon  *microphone* | | Taschenlampe  *flashlight* |
| Zigarettenschachtel *cigarette box* | Federball  *shuttlecock* | Fliegenklatsche  *fly swatter* | Sieb  *colander* | | Tennischläger  *tennis racket* |
| Nähmaschine  *sewing machine* | Schwimmweste  *life jacket* | Wischmop  *mop* | Löffel  *spoon* | | Paddel  *oar* |
| Motorsense  *grass mower* | Bleistiftspitzer  *pencil sharpener* | Klebestift  *gluestick* | USB-Stick  *memory stick* | | Radiergummi  *eraser* |
| Heizkörper  *radiator* | Bettvorleger  *rug* | Baseballschläger  *baseball bat* | Kochlöffel  *cooking spatula* | | Teppichklopfer  *carpet beater* |
| Skistock  *ski pole* | Trompete  *trumpet* | Blasebalg  *bellows* | Fächer  *Chinese fan* | | Accordeon  *accordion* |
| Teebeutel  *teabag* | Videokamera  *video camera* | Haarspray  *hairspray* | Walkman  *walkman* | | Fotokamera  *camera* |
| Serviette  *napkin* | Schraubenschlüssel  *wrench* | Fahrradbremse  *bike brakes* | Wünschelrute  *dowsing rods* | | Zange  *pliers* |
| Kaffeetasse  *coffee cup* | Schubkarre  *wheelbarrow* | Staubsauger  *vacuum cleaner* | Schneeschieber  *snow shovel* | | Schaufel  *shovel* |
| Funkgeräte  *walkie-talkie* | Zielscheibe  *dartboard* | Papierflieger  *paper plane* | Spritze  *syringe* | | Dartpfeil  *dart* |
| Blutdruckmesser  *blood pressure monitor* | Weinglas  *wine glass* | Türknopf  *doorknob* | Spiralfeder  *coil spring* | | Korkenzieher  *corkscrew* |
| Ladegerät  *battery charger* | Dübel  *rawlplug* | Haustürschlüssel  *housekey* | Lötkolben  *soldering iron* | | Schraubenzieher  *screwdriver* |
| Stehlampe  *floor lamp* | Flaschenetikett  *bottle label* | Wasserhahn  *faucet* | Münze  *coin* | | Schraubverschluss  *bottlecap* |
| Handschuh  *glove* | Heizdraht  *heating coil* | Toilettenspülung  *toilet flush* | Kofferradio  *radio* | | Toaster  *toaster* |
| Schlagzeug  *drumkit* | Leuchtdiode  *LED* | Lautstärkeregler  *volume knob* | Frühstücksei  *egg* | | Glühbirne  *lightbulb* |
| Gartenschlauch  *garden hose* | Pizza  *pizza* | Schuhbürste  *shoebrush* | Gitterrost  *wire grate* | | Käsereibe  *cheese grater* |
| Klappbett  *folding bed* | Maßband  *tape measure* | Feuerzeug  *lighter* | Barometer  *barometer* | | Stoppuhr  *stopwatch* |
| Hundeleine  *dog leash* | Waschmaschine  *washing machine* | Cellobogen  *cello bow* | Maurerkelle  *trowel* | | Bügeleisen  *clothes iron* |
| Registrierkasse  *cash register* | Gummi  *eraser* | Trillerpfeife  *whistle* | Motorradhelm  *helmet* | | Luftballon  *balloon* |

**Appendix 2.** Norming data for the four conditions rated along semantic, action, and visual relatedness on a scale of 1 (not related) to 7 (highly related). Standard deviation values within parentheses.

|  | **semantic relatedness** | **action relatedness** | **visual relatedness** |
| --- | --- | --- | --- |
| **unrelated** | 1.3 (*0.6*) | 1.3 (*0.6*) | 1.2 (*0.6*) |
| **semantic** | 6.0 (*1.0*) | 1.9 (*1.1*) | 1.7 (*1.0*) |
| **action** | 1.7 (*1.0*) | 5.9 (*1.0*) | 1.7 (*1.0*) |
| **visual** | 1.6 (*1.0*) | 1.6 (*1.0*) | 5.9 (*1.1*) |

**Appendix 3.** Mean word frequencies and lengths of primes and targets for the four conditions. Standard deviation values within parentheses. Note that the same targets are used across conditions.

|  | **prime word** | | |  | **target word** | |
| --- | --- | --- | --- | --- | --- | --- |
|  | **frequency** | **length** |  |  | **frequency** | **length** |
| **unrelated** | 1.08 (*0.6*) | 10.8 (*2.8*) |  |  | 1.36 (*0.5*) | 10.3 (*2.9*) |
| **semantic** | 1.28 (*0.7*) | 10.4 (*3.4*) |  |  |  |  |
| **action** | 1.20 (*0.6*) | 11.7 (*2.9*) |  |  |  |  |
| **visual** | 1.17 (*0.7*) | 9.5 (*2.6*) |  |  |  |  |
